# Supplementary material for: Associations between physical activity and CVD-related metabolomic and proteomic biomarkers
Source: PLoS One. 2025 Jun 11;20(6):e0325720. doi: 10.1371/journal.pone.0325720 (PMC12157240; doi:10.1371/journal.pone.0325720)
Supplement: S1 Table — (DOCX) [file pone.0325720.s001.docx]

Supplementary table 5.

| PA class | Non-significant metabolomic markers | Non-significant proteomic  markers |
| --- | --- | --- |
| SED  (Met n=81)  (Prot n=158) | \| AcAce \| \| --- \| \| Ace \| \| Alb \| \| ApoB \| \| bOHBut \| \| Cit \| \| DHA \| \| DHAFA \| \| Dkk1 \| \| EpCAM \| \| EstC \| \| FAw3 \| \| FAw3FA \| \| FAw6 \| \| FreeC \| \| Gln \| \| HDL3C \| \| HDLTG \| \| His \| \| IDLC \| \| IDLCE \| \| IDLFC \| \| IDLL \| \| IDLP \| \| IDLPL \| \| LA \| \| LAFA \| \| LDLC \| \| LDLD \| \| LDLTG \| \| LHDLTG \| \| LLDLC \| \| LLDLCE \| \| LLDLFC \| \| LLDLL \| \| LLDLP \| \| LLDLPL \| \| LLDLTG \| \| MHDLTG \| \| MLDLC \| \| MLDLCE \| \| MLDLFC \| \| MLDLL \| \| MLDLP \| \| MLDLPL \| \| MLDLTG \| \| MUFA \| \| PC \| \| PUFA \| \| RemnantC \| \| SerumC \| \| SFA \| \| SFAFA \| \| SHDLC \| \| SHDLCE \| \| SHDLFC \| \| SHDLL \| \| SHDLP \| \| SHDLPL \| \| SLDLC \| \| SLDLCE \| \| SLDLFC \| \| SLDLL \| \| SLDLP \| \| SLDLPL \| \| SLDLTG \| \| SM \| \| TotCho \| \| TotFA \| \| TotPG \| \| Tyr \| \| XLHDLC \| \| XLHDLCE \| \| XLHDLTG \| \| XSVLDLC \| \| XSVLDLCE \| \| XSVLDLFC \| \| XSVLDLL \| \| XSVLDLPL \| \| XXLVLDLFC \| \| XXLVLDLPL \| | \| \| ACE2 \| \| --- \| \| ADAMTS13 \| \| AGRP \| \| ALCAM \| \| AMBP \| \| ANG1 \| \| APN \| \| AXL \| \| AZU1 \| \| BLMhydrolase \| \| BMP6 \| \| BNP \| \| BOC \| \| CASP3 \| \| CCL15 \| \| CCL16 \| \| CCL17 \| \| CCL24 \| \| CCL3 \| \| CD163 \| \| CD40L \| \| CD84 \| \| CD93 \| \| CDH5 \| \| CHI3L1 \| \| CHIT1 \| \| CNTN1 \| \| COL1A1 \| \| CPA1 \| \| CPB1 \| \| CSTB \| \| CTRC \| \| CTSD \| \| CTSL1 \| \| CXCL1 \| \| CXCL16 \| \| DCN \| \| DECR1 \| \| DLK1 \| \| EPHB4 \| \| FABP2 \| \| FAS \| \| FS \| \| Gal3 \| \| Gal4 \| \| GDF15 \| \| GDF2 \| \| GH \| \| GIF \| \| GLO1 \| \| GP6 \| \| GRN \| \| HBEGF \| \| HO1 \| \| hOSCAR \| \| HSP27 \| \| ICAM2 \| \| IDUA \| \| IGFBP1 \| \| IGFBP2 \| \| IGFBP7 \| \| IgGFcreceptorIIb \| \| IL16 \| \| IL17D \| \| IL17RA \| \| IL18 \| \| IL18BP \| \| IL1RL2 \| \| IL1RT1 \| \| IL1RT2 \| \| IL27 \| \| IL2RA \| \| IL4RA \| \| IL6 \| \| IL6RA \| \| ITGB1BP2 \| \| JAMA \| \| KIM1 \| \| KLK6 \| \| LDLreceptor \| \| LOX1 \| \| LTBR \| \| MARCO \| \| MB \| \| MCP1 \| \| MEPE \| \| MERTK \| \| MMP12 \| \| MMP2 \| \| MMP3 \| \| MMP7 \| \| MMP9 \| \| MPO \| \| NEMO \| \| Notch3 \| \| NTproBNP \| \| OPG \| \| OPN \| \| PAI \| \| PAPPA \| \| PAR1 \| \| PARP1 \| \| PCSK9 \| \| PDGFsubunitA \| \| PDGFsubunitB \| \| PDL2 \| \| PECAM1 \| \| PGF \| \| PGLYRP1 \| \| PIgR \| \| PLC \| \| PON3 \| \| PRELP \| \| PRSS27 \| \| PRSS8 \| \| PRTN3 \| \| PSGL1 \| \| PSPD \| \| PTX3 \| \| RAGE \| \| RARRES2 \| \| REN \| \| SCF \| \| SCGB3A2 \| \| SELE \| \| SELP \| \| SERPINA12 \| \| SHPS1 \| \| SLAMF7 \| \| SOD2 \| \| SORT1 \| \| SPON1 \| \| SRC \| \| ST2 \| \| STK4 \| \| TF \| \| TFF3 \| \| TFPI \| \| TGM2 \| \| THBS2 \| \| THPO \| \| TIE2 \| \| TLT2 \| \| TM \| \| TNFRSF10A \| \| TNFRSF10C \| \| TNFRSF13B \| \| TNFRSF14 \| \| TNFSF13B \| \| tPA \| \| TR \| \| TRAILR2 \| \| TRAP \| \| uPA \| \| UPAR \| \| VEGFD \| \| VSIG2 \| \| vWF \| \| \| --- \| --- \| --- \| --- \| --- \| --- \| --- \| --- \| --- \| --- \| --- \| --- \| --- \| --- \| --- \| --- \| --- \| --- \| --- \| --- \| --- \| --- \| --- \| --- \| --- \| --- \| --- \| --- \| --- \| --- \| --- \| --- \| --- \| --- \| --- \| --- \| --- \| --- \| --- \| --- \| --- \| --- \| --- \| --- \| --- \| --- \| --- \| --- \| --- \| --- \| --- \| --- \| --- \| --- \| --- \| --- \| --- \| --- \| --- \| --- \| --- \| --- \| --- \| --- \| --- \| --- \| --- \| --- \| --- \| --- \| --- \| --- \| --- \| --- \| --- \| --- \| --- \| --- \| --- \| --- \| --- \| --- \| --- \| --- \| --- \| --- \| --- \| --- \| --- \| --- \| --- \| --- \| --- \| --- \| --- \| --- \| --- \| --- \| --- \| --- \| --- \| --- \| --- \| --- \| --- \| --- \| --- \| --- \| --- \| --- \| --- \| --- \| --- \| --- \| --- \| --- \| --- \| --- \| --- \| --- \| --- \| --- \| --- \| --- \| --- \| --- \| --- \| --- \| --- \| --- \| --- \| --- \| --- \| --- \| --- \| --- \| --- \| --- \| --- \| --- \| --- \| --- \| --- \| --- \| --- \| --- \| --- \| --- \| --- \| --- \| --- \| --- \| --- \| --- \| --- \| --- \| --- \| --- \| --- \| |
|  |  |  |
| Prol SED  (Met n=135)  (Prot n=170) | \| AcAce \| \| --- \| \| Ace \| \| Ala \| \| ApoB \| \| ApoBApoA1 \| \| bOHBut \| \| Cit \| \| DHA \| \| DHAFA \| \| Dkk1 \| \| EpCAM \| \| EstC \| \| FAw3 \| \| FAw3FA \| \| FAw6 \| \| FAw6FA \| \| FreeC \| \| Gln \| \| Gp \| \| HDL3C \| \| HDLD \| \| HDLTG \| \| His \| \| IDLC \| \| IDLCE \| \| IDLFC \| \| IDLL \| \| IDLP \| \| IDLPL \| \| IDLTG \| \| Ile \| \| LA \| \| LAFA \| \| LDLC \| \| LDLD \| \| LDLTG \| \| Leu \| \| LHDLFC \| \| LHDLTG \| \| LLDLC \| \| LLDLCE \| \| LLDLFC \| \| LLDLL \| \| LLDLP \| \| LLDLPL \| \| LLDLTG \| \| LVLDLC \| \| LVLDLCE \| \| LVLDLFC \| \| LVLDLL \| \| LVLDLP \| \| LVLDLPL \| \| LVLDLTG \| \| MHDLTG \| \| MLDLC \| \| MLDLCE \| \| MLDLFC \| \| MLDLL \| \| MLDLP \| \| MLDLPL \| \| MLDLTG \| \| MUFA \| \| MUFAFA \| \| MVLDLC \| \| MVLDLCE \| \| MVLDLFC \| \| MVLDLL \| \| MVLDLP \| \| MVLDLPL \| \| MVLDLTG \| \| PC \| \| PUFA \| \| PUFAFA \| \| RemnantC \| \| SerumC \| \| SerumTG \| \| SFA \| \| SFAFA \| \| SHDLC \| \| SHDLCE \| \| SHDLFC \| \| SHDLL \| \| SHDLP \| \| SHDLPL \| \| SHDLTG \| \| SLDLC \| \| SLDLCE \| \| SLDLFC \| \| SLDLL \| \| SLDLP \| \| SLDLPL \| \| SLDLTG \| \| SM \| \| SVLDLC \| \| SVLDLFC \| \| SVLDLL \| \| SVLDLP \| \| SVLDLPL \| \| TotCho \| \| TotFA \| \| TotPG \| \| Tyr \| \| UnSat \| \| Val \| \| VLDLC \| \| VLDLD \| \| VLDLTG \| \| XLHDLC \| \| XLHDLCE \| \| XLHDLFC \| \| XLHDLL \| \| XLHDLP \| \| XLHDLPL \| \| XLHDLTG \| \| XLVLDLC \| \| XLVLDLCE \| \| XLVLDLFC \| \| XLVLDLL \| \| XLVLDLP \| \| XLVLDLPL \| \| XLVLDLTG \| \| XSVLDLC \| \| XSVLDLCE \| \| XSVLDLFC \| \| XSVLDLL \| \| XSVLDLP \| \| XSVLDLPL \| \| XSVLDLTG \| \| XXLVLDLC \| \| XXLVLDLCE \| \| XXLVLDLFC \| \| XXLVLDLL \| \| XXLVLDLP \| \| XXLVLDLPL \| \| XXLVLDLTG \| | \| ACE2 \| \| --- \| \| ADAMTS13 \| \| AGRP \| \| ALCAM \| \| AMBP \| \| ANG1 \| \| APN \| \| AXL \| \| AZU1 \| \| BLMhydrolase \| \| BMP6 \| \| BNP \| \| BOC \| \| CASP3 \| \| CCL15 \| \| CCL16 \| \| CCL17 \| \| CCL24 \| \| CCL3 \| \| CD163 \| \| CD4 \| \| CD40L \| \| CD84 \| \| CD93 \| \| CDH5 \| \| CEACAM8 \| \| CHI3L1 \| \| CHIT1 \| \| CNTN1 \| \| COL1A1 \| \| CPA1 \| \| CPB1 \| \| CSTB \| \| CTRC \| \| CTSD \| \| CTSZ \| \| CXCL1 \| \| CXCL16 \| \| DCN \| \| DECR1 \| \| DLK1 \| \| EPHB4 \| \| FABP2 \| \| FAS \| \| FGF21 \| \| FS \| \| Gal3 \| \| Gal4 \| \| GDF15 \| \| GDF2 \| \| GH \| \| GIF \| \| GLO1 \| \| GP6 \| \| GRN \| \| HAOX1 \| \| HBEGF \| \| HO1 \| \| hOSCAR \| \| HSP27 \| \| ICAM2 \| \| IDUA \| \| IGFBP1 \| \| IGFBP2 \| \| IGFBP7 \| \| IgGFcreceptorIIb \| \| IL16 \| \| IL17D \| \| IL17RA \| \| IL18 \| \| IL18BP \| \| IL1ra \| \| IL1RL2 \| \| IL1RT1 \| \| IL1RT2 \| \| IL27 \| \| IL2RA \| \| IL4RA \| \| IL6 \| \| IL6RA \| \| ITGB1BP2 \| \| ITGB2 \| \| JAMA \| \| KIM1 \| \| KLK6 \| \| LDLreceptor \| \| LOX1 \| \| LPL \| \| LTBR \| \| MARCO \| \| MB \| \| **MCP1** \| \| MEPE \| \| MERTK \| \| MMP12 \| \| MMP2 \| \| MMP3 \| \| MMP7 \| \| MMP9 \| \| MPO \| \| NEMO \| \| Notch3 \| \| NTproBNP \| \| OPG \| \| OPN \| \| PAI \| \| PAPPA \| \| PAR1 \| \| PARP1 \| \| PCSK9 \| \| PDGFsubunitA \| \| PDGFsubunitB \| \| PDL2 \| \| PECAM1 \| \| PGF \| \| PGLYRP1 \| \| PIgR \| \| PLC \| \| PON3 \| \| PRELP \| \| PRSS27 \| \| PRSS8 \| \| PRTN3 \| \| PSGL1 \| \| PSPD \| \| PTX3 \| \| RAGE \| \| RARRES2 \| \| REN \| \| RETN \| \| SCF \| \| SCGB3A2 \| \| SELE \| \| SELP \| \| SERPINA12 \| \| SHPS1 \| \| SLAMF7 \| \| SOD2 \| \| SORT1 \| \| SPON1 \| \| SRC \| \| ST2 \| \| STK4 \| \| SVLDLTG \| \| TF \| \| TFF3 \| \| TFPI \| \| TGM2 \| \| TGPG \| \| THBS2 \| \| THPO \| \| TIE2 \| \| TIMP4 \| \| TLT2 \| \| TM \| \| TNFR2 \| \| TNFRSF10A \| \| TNFRSF10C \| \| TNFRSF13B \| \| TNFRSF14 \| \| TNFSF13B \| \| tPA \| \| TR \| \| TRAP \| \| uPA \| \| UPAR \| \| VEGFD \| \| VSIG2 \| \| vWF \| \| XCL1 \| |
|  |  |  |
| MVPA  (Met n=104)  (Prot n=155) | \| AcAce \| \| --- \| \| Ace \| \| Alb \| \| ApoA1 \| \| ApoB \| \| ApoBApoA1 \| \| Cit \| \| Crea \| \| DHA \| \| DHAFA \| \| Dkk1 \| \| EpCAM \| \| EstC \| \| FAw3 \| \| FAw3FA \| \| FAw6 \| \| FreeC \| \| Gln \| \| HDL2C \| \| HDL3C \| \| HDLC \| \| HDLD \| \| His \| \| IDLC \| \| IDLCE \| \| IDLFC \| \| IDLL \| \| IDLP \| \| IDLPL \| \| IDLTG \| \| LA \| \| Lac \| \| LDLC \| \| LDLD \| \| LDLTG \| \| Leu \| \| LHDLC \| \| LHDLCE \| \| LHDLFC \| \| LHDLL \| \| LHDLP \| \| LHDLPL \| \| LHDLTG \| \| LLDLC \| \| LLDLCE \| \| LLDLFC \| \| LLDLL \| \| LLDLP \| \| LLDLPL \| \| LLDLTG \| \| MHDLC \| \| MHDLCE \| \| MHDLFC \| \| MHDLL \| \| MHDLP \| \| MHDLPL \| \| MLDLC \| \| MLDLCE \| \| MLDLFC \| \| MLDLL \| \| MLDLP \| \| MLDLPL \| \| MLDLTG \| \| PC \| \| Phe \| \| PUFA \| \| RemnantC \| \| SerumC \| \| SFA \| \| SFAFA \| \| SHDLC \| \| SHDLCE \| \| SHDLFC \| \| SHDLL \| \| SHDLP \| \| SHDLPL \| \| SLDLC \| \| SLDLCE \| \| SLDLFC \| \| SLDLL \| \| SLDLP \| \| SLDLPL \| \| SM \| \| SVLDLC \| \| SVLDLCE \| \| TotCho \| \| TotFA \| \| TotPG \| \| Tyr \| \| UnSat \| \| Val \| \| XLHDLC \| \| XLHDLCE \| \| XLHDLFC \| \| XLHDLL \| \| XLHDLP \| \| XLHDLPL \| \| XLHDLTG \| \| XSVLDLC \| \| XSVLDLCE \| \| XSVLDLFC \| \| XSVLDLL \| \| XSVLDLP \| \| XSVLDLPL \| | \| ACE2 \| \| --- \| \| ADAMTS13 \| \| ADM \| \| AGRP \| \| ALCAM \| \| AMBP \| \| ANG1 \| \| APN \| \| AXL \| \| AZU1 \| \| BLMhydrolase \| \| BMP6 \| \| BNP \| \| BOC \| \| CA5A \| \| CASP3 \| \| CCL15 \| \| CCL16 \| \| CCL17 \| \| CCL24 \| \| CCL3 \| \| CD163 \| \| CD4 \| \| CD40L \| \| CD84 \| \| CD93 \| \| CDH5 \| \| CHIT1 \| \| CNTN1 \| \| COL1A1 \| \| CPA1 \| \| CPB1 \| \| CSTB \| \| CTRC \| \| CTSD \| \| CTSL1 \| \| CTSZ \| \| CXCL1 \| \| CXCL16 \| \| DCN \| \| DECR1 \| \| DLK1 \| \| EGFR \| \| EPHB4 \| \| FABP2 \| \| FABP4 \| \| FAS \| \| FGF23 \| \| FS \| \| Gal3 \| \| Gal4 \| \| Gal9 \| \| GDF2 \| \| GH \| \| GIF \| \| GLO1 \| \| GP6 \| \| GRN \| \| HAOX1 \| \| HBEGF \| \| HO1 \| \| hOSCAR \| \| HSP27 \| \| ICAM2 \| \| IDUA \| \| IGFBP1 \| \| IGFBP2 \| \| IGFBP7 \| \| IgGFcreceptorIIb \| \| IL16 \| \| IL17D \| \| IL17RA \| \| IL18 \| \| IL18BP \| \| IL1ra \| \| IL1RL2 \| \| IL1RT1 \| \| IL1RT2 \| \| IL27 \| \| IL2RA \| \| IL4RA \| \| IL6RA \| \| ITGB1BP2 \| \| JAMA \| \| KIM1 \| \| KLK6 \| \| LDLreceptor \| \| LTBR \| \| MARCO \| \| MB \| \| MCP1 \| \| MEPE \| \| MERTK \| \| MMP2 \| \| MMP3 \| \| MPO \| \| NEMO \| \| Notch3 \| \| NTproBNP \| \| OPG \| \| OPN \| \| PAI \| \| PAPPA \| \| PAR1 \| \| PARP1 \| \| PCSK9 \| \| PDGFsubunitA \| \| PDGFsubunitB \| \| PDL2 \| \| PECAM1 \| \| PIgR \| \| PLC \| \| PRELP \| \| PRSS27 \| \| PSGL1 \| \| PSPD \| \| PTX3 \| \| RAGE \| \| SCF \| \| SCGB3A2 \| \| SELE \| \| SELP \| \| SERPINA12 \| \| SHPS1 \| \| SLAMF7 \| \| SOD2 \| \| SORT1 \| \| SPON1 \| \| SPON2 \| \| SRC \| \| ST2 \| \| STK4 \| \| TF \| \| TFPI \| \| TGM2 \| \| THBS2 \| \| THPO \| \| TIE2 \| \| TIMP4 \| \| TLT2 \| \| TM \| \| TNFR1 \| \| TNFR2 \| \| TNFRSF10A \| \| TNFRSF10C \| \| TNFRSF11A \| \| TNFRSF13B \| \| TNFRSF14 \| \| TR \| \| TRAP \| \| uPA \| \| VEGFD \| \| VSIG2 \| \| vWF \| \| XCL1 \| |

*Note:* for full name and description of each biomarker, please refer to https://scapisportal.it.gu.se/portal/variables
