# Supplementary material for: Associations between physical activity and CVD-related metabolomic and proteomic biomarkers
Source: PLoS One. 2025 Jun 11;20(6):e0325720. doi: 10.1371/journal.pone.0325720 (PMC12157240; doi:10.1371/journal.pone.0325720)
Supplement: S2 Table — (DOCX) [file pone.0325720.s002.docx]

***Supplementary table 1.***

| Marker | Beta Female | Beta Male | Interaction p-value |  |
| --- | --- | --- | --- | --- |
| *Metabolomic* |  |  |  |  |
| FAw6FA | -0,00828 | -0,01934 | 0,040 |  |
| Ile | 5,64E-05 | 0,000123 | 0,024 |  |
| Leu | 3,78E-05 | 0,000111 | 0,029 |  |
| LHDLC | -0,00196 | -0,00114 | 0,043 |  |
| LHDLCE | -0,00151 | -0,00087 | 0,039 |  |
| TGPG | 0,001036 | 0,002141 | 0,036 |  |
| XLHDLC | -0,00086 | -0,00025 | 0,006 |  |
| XLHDLFC | -0,00027 | -8,2E-05 | 0,005 |  |
| XLHDLL | -0,00197 | -0,00066 | 0,007 |  |
| XLHDLP | -1,9E-09 | -6,5E-10 | 0,007 |  |
| XLHDLPL | -0,00108 | -0,00043 | 0,014 |  |
| XLVLDLFC | 1,01E-05 | 3,48E-05 | 0,041 |  |
| XXLVLDLL | 3,7E-05 | 0,000143 | 0,034 |  |
| XXLVLDLP | 1,71E-13 | 6,65E-13 | 0,034 |  |
| XXLVLDLTG | 2,33E-05 | 9,86E-05 | 0,030 |  |
| *Proteomic*  LEP | 0,009375 | 0,01423 | 0,041 |  |
| RETN | 0,001667 | 0,004467 | 0,028 |  |
| SPON2 | 0,000321 | 0,001732 | 0,006 |  |
