# Supplementary material for: Associations between physical activity and CVD-related metabolomic and proteomic biomarkers
Source: PLoS One. 2025 Jun 11;20(6):e0325720. doi: 10.1371/journal.pone.0325720 (PMC12157240; doi:10.1371/journal.pone.0325720)
Supplement: S3 Table — (DOCX) [file pone.0325720.s003.docx]

***Supplementary table 2.***

| Marker | Beta Female | Beta Male | Interaction p-value |
| --- | --- | --- | --- |
| *Metabolomic* |  |  |  |
| XLHDLPL | 0,001672 | -0,00029 | 0,014 |
| XLHDLL | 0,003045 | -0,00057 | 0,014 |
| XLHDLP | 2,97E-09 | -6,1E-10 | 0,013 |
| XLHDLFC | 0,000427 | -5,9E-05 | 0,014 |
| *Proteomic* |  |  |  |
| ITGB2 | 0,011152 | 0,004513 | 0,035 |
| XLHDLC | 0,001389 | -0,00015 | 0,023 |
