# Supplementary material for: Associations between physical activity and CVD-related metabolomic and proteomic biomarkers
Source: PLoS One. 2025 Jun 11;20(6):e0325720. doi: 10.1371/journal.pone.0325720 (PMC12157240; doi:10.1371/journal.pone.0325720)
Supplement: S4 Table — (DOCX) [file pone.0325720.s004.docx]

**Supplementary table 3.**

| Marker | Low WC | High WC | P-value |
| --- | --- | --- | --- |
| *Metabolomic* |  |  |  |
| Crea | 0,0000702 | 0,0001200 | 0,008 |
| Lac | 0,0010631 | 0,0031789 | 0,008 |
| MVLDLCE | 0,0003381 | 0,0000939 | 0,015 |
| Phe | 0,0000172 | 0,0000622 | 0,005 |
| SVLDLC | 0,0005823 | 0,0001078 | 0,006 |
| SVLDLCE | 0,0003825 | 0,0000323 | 0,002 |
| SVLDLFC | 0,0001998 | 0,0000755 | 0,049 |
| SVLDLL | 0,0015910 | 0,0006568 | 0,043 |
| SVLDLPL | 0,0003199 | 0,0001240 | 0,047 |
| UnSat | -0,0001349 | -0,0004063 | 0,023 |
| VLDLC | 0,0018335 | 0,0005005 | 0,021 |
| XSVLDLP | 5,8489E-11 | 3,5847E-12 | 0,015 |
| *Proteomic* |  |  |  |
| ADM | 0,0040797 | 0,0062739 | 0,039 |
| FABP4 | 0,0041626 | 0,0096057 | 0,002 |
| HAOX1 | 0,0029288 | 0,0107057 | 0,032 |
| PARP1 | 0,0013543 | 0,0037231 | 0,035 |
| SPON2 | 0,0004859 | 0,0017364 | 0,015 |
| TIMP4 | 0,0007736 | 0,0034717 | 0,022 |
| TNFRSF11A | 0,0027073 | 0,0057772 | 0,005 |
