# Supplementary material for: Associations between physical activity and CVD-related metabolomic and proteomic biomarkers
Source: PLoS One. 2025 Jun 11;20(6):e0325720. doi: 10.1371/journal.pone.0325720 (PMC12157240; doi:10.1371/journal.pone.0325720)
Supplement: S5 Table — (DOCX) [file pone.0325720.s005.docx]

**Supplementary table 4.**

| Marker | Low WC | High WC | P-value |
| --- | --- | --- | --- |
| *Metabolomic* |  |  |  |
| ApoBApoA1 | -0,0022772 | 0,0001045 | 0,031 |
| SVLDLC | -0,0009240 | 0,0002305 | 0,030 |
| SVLDLCE | -0,0006018 | 0,0003724 | 0,006 |
| UnSat | 0,0000065 | 0,0008697 | 0,019 |
| XSVLDLP | -1,077E-10 | 7,189E-11 | 0,010 |
| Proteomic |  |  |  |
| CTSZ | -0,0002191 | -0,0092904 | 0,008 |
| HAOX1 | 0,0050655 | -0,0172056 | 0,047 |
