# Supplementary figures and images for: Associations between physical activity and CVD-related metabolomic and proteomic biomarkers
Source: PLoS One. 2025 Jun 11;20(6):e0325720. doi: 10.1371/journal.pone.0325720 (PMC12157240; doi:10.1371/journal.pone.0325720)

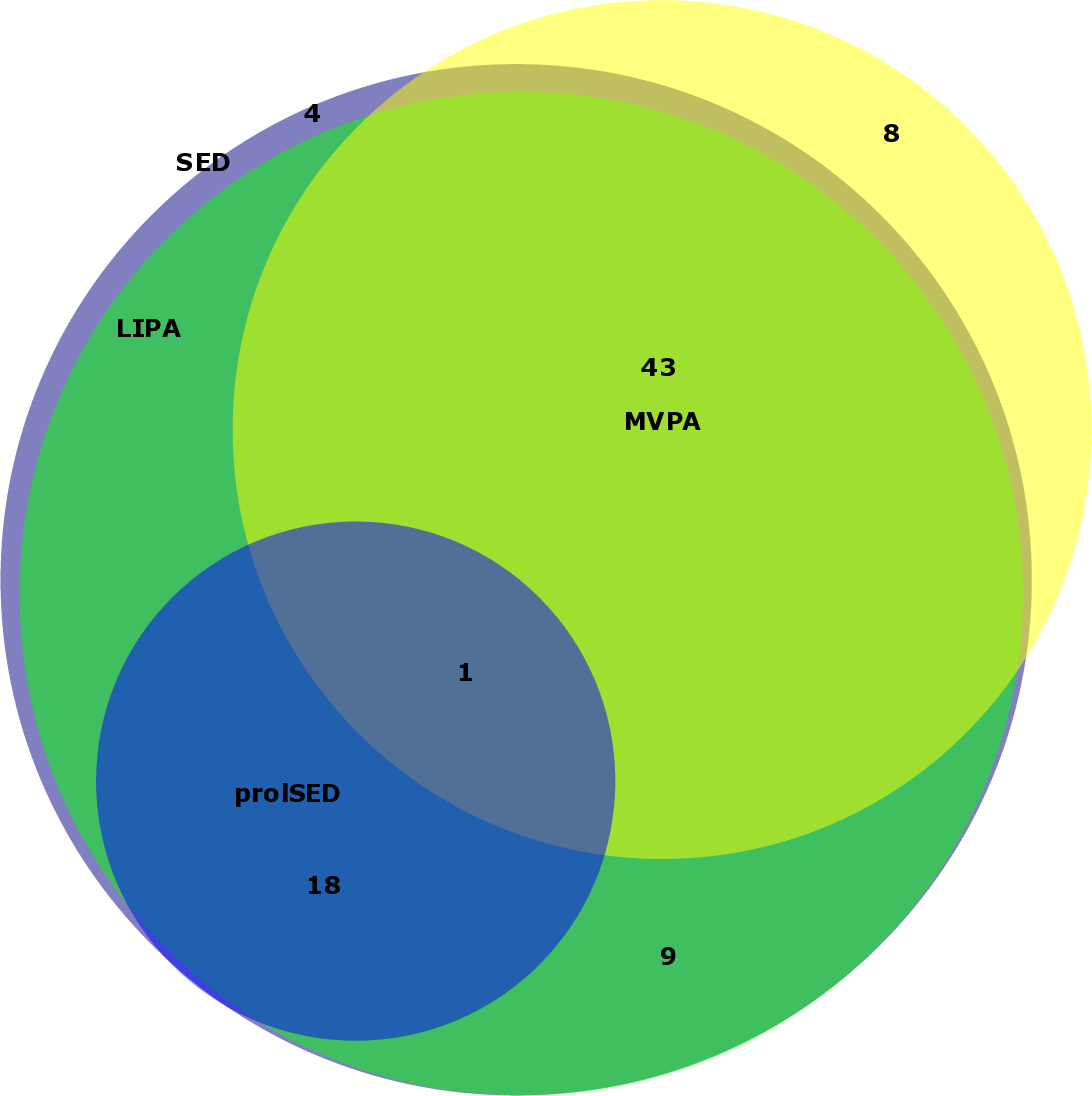

Supplement: S1 Fig — SED encapsuled all biomarkers related to LIPA. (TIF) [file pone.0325720.s006.tif]

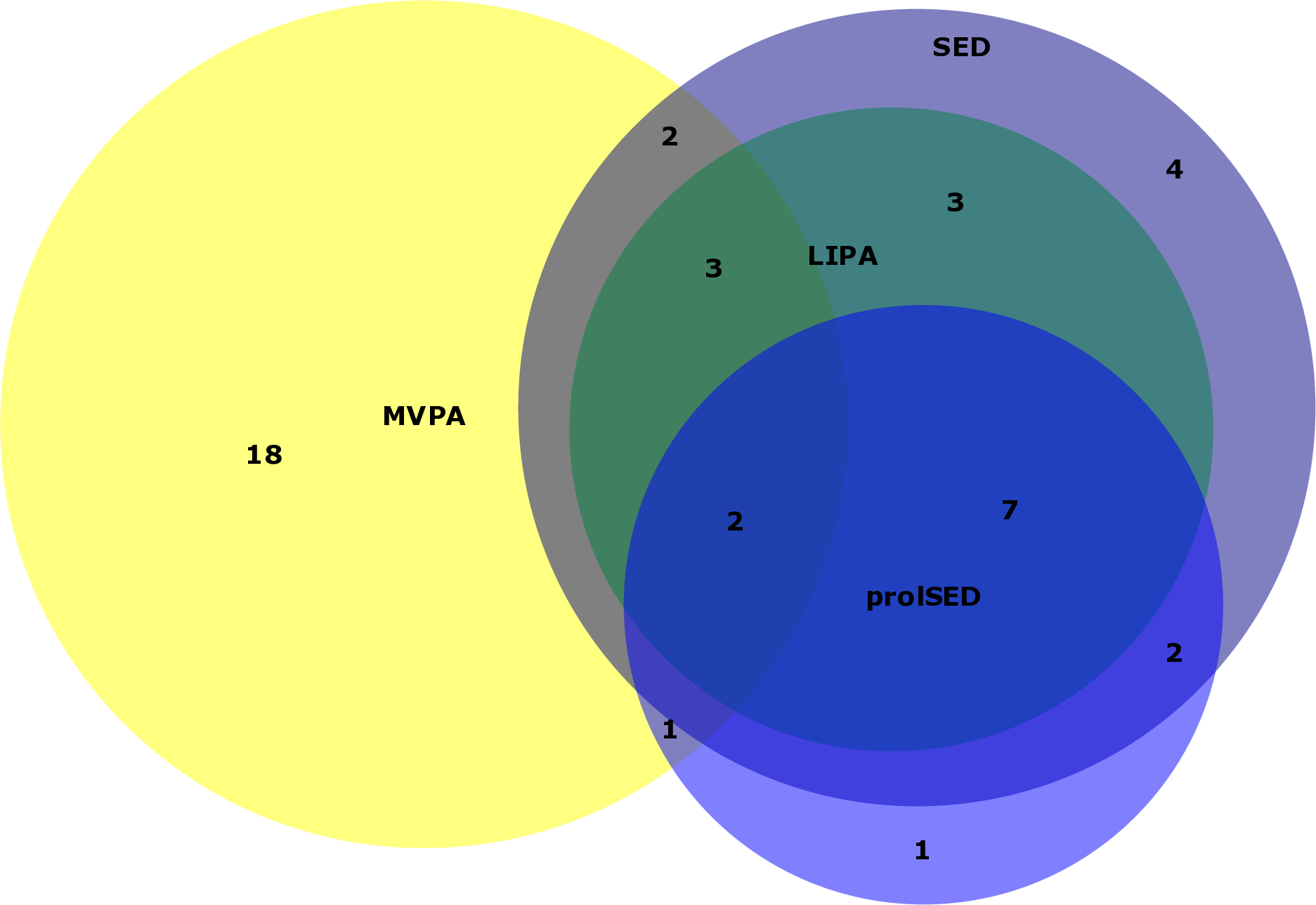

Supplement: S2 Fig — SED encapsuled all biomarkers related to LIPA. (TIF) [file pone.0325720.s007.tif]

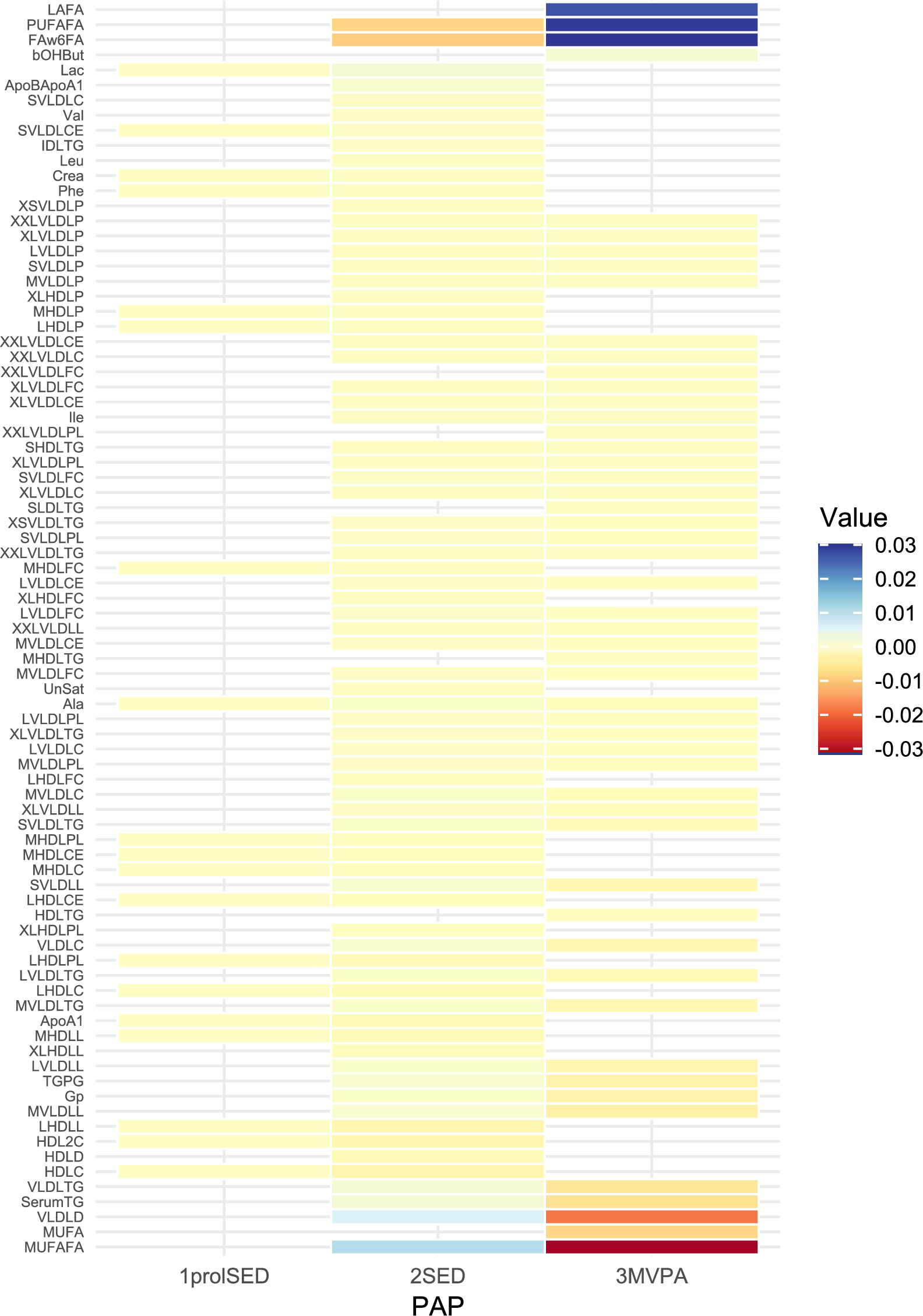

Supplement: S3 Fig — (TIF) [file pone.0325720.s008.tif]

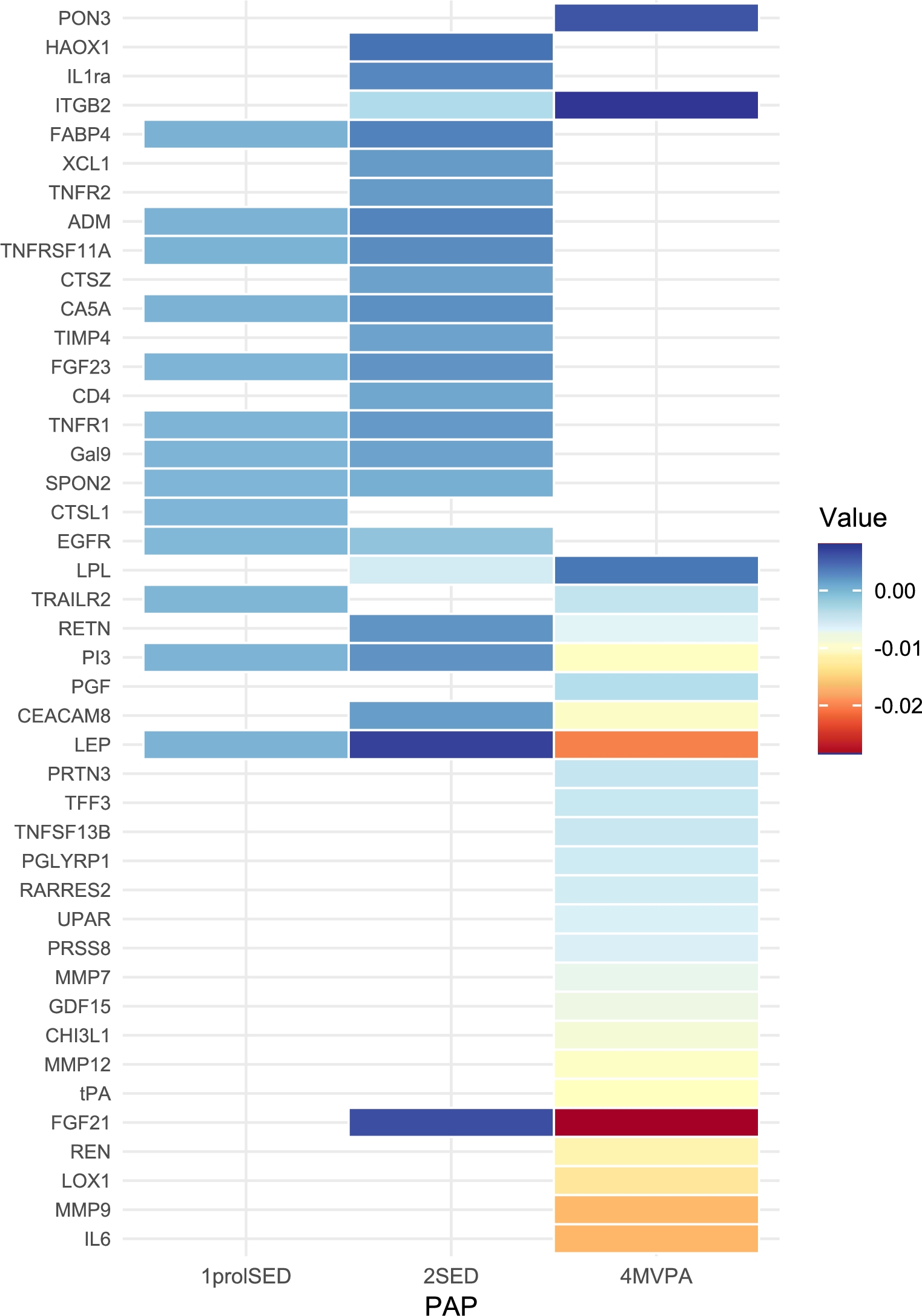

Supplement: S4 Fig — (TIF) [file pone.0325720.s009.tif]
