# Supplementary material for: Associations between physical activity and CVD-related metabolomic and proteomic biomarkers
Source: PLoS One. 2025 Jun 11;20(6):e0325720. doi: 10.1371/journal.pone.0325720 (PMC12157240; doi:10.1371/journal.pone.0325720)
Supplement: Attachment — Submitted filename: Ekblom_Response to reviewers.docx [file pone.0325720.s012.docx]

| Journal requirements |  |  |
| --- | --- | --- |
| Comment | Author response | Alterations |
| 1. Please ensure that your manuscript meets PLOS ONE's style requirements, including those for file naming. The PLOS ONE style templates can be found at | We have compared requirements including naming. |  |
| 2. Thank you for stating the following financial disclosure:  [ÖE was funded by Livförsäkringsbolaget Skandia, Risk&Hälsa.]. At this time, please address the following queries: |  |  |
|  |  |  |
| a) Please clarify the sources of funding (financial or material support) for your study. List the grants or organizations that supported your study, including funding received from your institution. | Thanks for pointing this out. This information has been added to the submission system. In the manuscript, this information has been moved from Acknowledgements to Funding. | Text updated on p 39, under "Acknowledgements" and "Funding" |
|  |  |  |
| b) State what role the funders took in the study. If the funders had no role in your study, please state: “The funders had no role in study design, data collection and analysis, decision to publish, or preparation of the manuscript.” | As the funding bodies did not have any such role, the statement has been added to the submission system and the manuscript. During the work on this paper, the SCAPIS office launched an altered standard wording for Acknowledgements, which has now been added | Text updated on p 39, under "Acknowledgements" and "Funding" |
|  |  |  |
| c) If any authors received a salary from any of your funders, please state which authors and which funders. | The main author was funded by a grant from Livförsäkringsbolaget Skandia in the form of salary. This has been added to the manuscript | Added text on p 39 under "Funding" |
|  |  |  |
| d) If you did not receive any funding for this study, please state: “The authors received no specific funding for this work.” |  |  |
|  |  |  |
| . We note that you have indicated that there are restrictions to data sharing for this study. For studies involving human research participant data or other sensitive data, we encourage authors to share de-identified or anonymized data. However, when data cannot be publicly shared for ethical reasons, we allow authors to make their data sets available upon request. For information on unacceptable data access restrictions, please see http://journals.plos.org/plosone/s/data-availability#loc-unacceptable-data-access-restrictions. |  |  |
| Before we proceed with your manuscript, please address the following prompts: |  |  |
| a) If there are ethical or legal restrictions on sharing a de-identified data set, please explain them in detail (e.g., data contain potentially identifying or sensitive patient information, data are owned by a third-party organization, etc.) and who has imposed them (e.g., a Research Ethics Committee or Institutional Review Board, etc.). Please also provide contact information for a data access committee, ethics committee, or other institutional body to which data requests may be sent. | Data used in this paper are a part of the SCAPIS database. Researcher aiming to obtain access to these data may apply for them to the data access committee at the SCAPIS office. The data access agreement between the authors and the SCAPIS hinders any direct sharing of data. Further, the ethical permissions for this paper do not allow for direct sharing of de-identified data. The data access committee can be reached at scapis@scapis.org, with more information at the website www.scapis.org. |  |
|  |  |  |
| b) If there are no restrictions, please upload the minimal anonymized data set necessary to replicate your study findings to a stable, public repository and provide us with the relevant URLs, DOIs, or accession numbers. Please see http://www.bmj.com/content/340/bmj.c181.long for guidelines on how to de-identify and prepare clinical data for publication. For a list of recommended repositories, please see https://journals.plos.org/plosone/s/recommended-repositories. You also have the option of uploading the data as Supporting Information files, but we would recommend depositing data directly to a data repository if possible. | n/a |  |
|  |  |  |
| Please update your Data Availability statement in the submission form accordingly. | Data Availability statement updated | Lägg in i SUBMISSION SYSTEM; The following text has been added: The General Data Protection Regulation (EU 2016/679) classifies de-identified versions of sensitive data that are sufficiently detailed to allow for re-identification as sensitive personal information. According to Swedish law (Law 2003:460 for ethical review of research involving humans), ethical permission is required to process such data. In accordance with Swedish legislation, the data can and will be made available to researchers who meet the criteria for access to confidential data, which includes obtaining their own ethics approval from the Swedish Ethical Review Authority (email: registrator@etikprovning.se; website: https://etikprovningsmyndigheten.se). Data applications can then be made by contacting SCAPIS (email: scapis@scapis.org; website: https://www.scapis.org/data-access/). |
|  |  |  |
| Reviewer #1 |  |  |
| I would like to congratulate the authors of developing this manuscript which is challenging due to the number of variables and sample size. It is an important information due to unclear association status between accelerometer-based measures of different physical activities and plasma metabolomic and proteomic biomarkers in a population-based study. Many relationship were anticipated, like the association of sedentary with HDL and higher activity level with VLDL and LDL. | We agree that the number of biomarkers in this paper is large, but also constitute a strength of the paper and that some of the relationships were expected. | None. |
|  |  |  |
| It was not clear from the manuscript whether diet plans were selected or advised to the participants and whether their diet could have any differences on the outcomes. | No diet plan was given, and diet was not controlled other than that subjects were in a fasted state during blood sampling. Food frequency questionnaires were used to survey the dietary habits of the participants. Data on macronutrient intake were included in the analyses. | Altered text on p 11, rows, 247-248 |
|  |  |  |
| It is worth to be mentioned by authors in the manuscript is the lack of detailed individuals health, medical conditions and therapies which might also affect outcomes like dyslipidemia and being on statin therapy as examples. | We agree that there are conditions that may be of relevance that we could not include, such as those mentioned. However, we chose to include overweight status, kidney function, diabetes status, along with behaviours such as diet and smoking. | We have included a paragraph on this in the limitations section, on p 37, rows 546-548 |
|  |  |  |
| There was two short titles for the manuscript, to review this. | We apologize, this has been adjusted. | The latter short title has been removed. |
|  |  |  |
| Reviewer #2 |  |  |
| The manuscript explores an important and timely topic, providing valuable insights into the associations between physical activity and cardiovascular biomarkers. While the study employs decent methodologies and presents significant findings, there are critical areas requiring attention. Addressing these issues will enhance the manuscript's impact. Below, I outline specific critiques for each section of the manuscript. | We thank the reviewer for the time taken to review our manuscript and the valuable input. |  |
|  |  |  |
| The title could be more concise. Consider removing redundant phrases like "assessed physical activity variables" to streamline it without losing clarity (e.g., "Associations between Physical Activity and CVD-related Biomarkers"). | Thank you for the input. We agree that the title can be perceived as bulky. | Title shortened for clarity. |
|  |  |  |
| Line 71–98: The terminology used (e.g., "mirroring," "robust relations") might be too technical for a general audience. Brief definitions or rephrasing could help. | We agree and have tried to clarify this further. | Alterations in results section of abstract. |
|  |  |  |
| The inclusion of exact numerical values for validated biomarkers might overwhelm the abstract's narrative. Consider summarizing these findings qualitatively. | We agree with the reviewer that this could be less detailed. However, we have discussed this and tried to express the results in overarching terms, without being too vague. We hope that this way of expressing the results might work in many readers minds. | none |
|  |  |  |
| Lines 100–133: There is an overreliance on citing older studies without critical analysis of gaps they leave. For example, it would be helpful to contrast self-reported physical activity data with accelerometer-based data more thoroughly. | We agree with the reviewer that this is most important. However, to our knowledge, there are no recent analysis that compare the predictive validity on biomarkers between self-reports and motion sensors. We therefore chose to base a discussion on this on an earlier paper and a few newer on similar topics. | Addition of discussion and references on rows 127-130. |
|  |  |  |
| The aim of the study (line 130–133) lacks a hypothesis-driven narrative. Stating a clear research question would enhance focus. | We agree with the reviewer that our aim is not hypothesis driven, but rather explorative. The large number of biomarkers studied makes it difficult to synthesise a single hypothesis, but based on studies presented in the background, it would be plausible to state that we believe that the relations would be different across PA classes. | On rows 165-166, we state We hypothesize that relations between PA and biomarkers are different across PA classes. |
|  |  |  |
| The description of accelerometer processing could be expanded to address potential biases introduced by the low-frequency extension filter. | The low-frequency extension (LFE) filter may to some extent affect time spent in SED or in prolSED, but not the other PA classes. The relations to the studied biomarkers are assumed to be more stable. | We have added a short discussion on this on rows 200-201. |
|  |  |  |
| No mention is made of how outliers or missing data were handled statistically (e.g., imputation methods). | We agree that this could be described in more detail. No imputation was applied as stated on row 235, but the text is now slightly more detailed. The extent of missing data is given under “Blood sampling and analysis”. | We state that “No imputation was applied for missing data.” on row 265-266. |
|  |  |  |
| Confounding variables (lines 203–214): While a comprehensive list is provided, there is no discussion on whether any were prioritized based on their theoretical significance. | We acknowledge this shortcoming to the text. The included independent variables all have their theoretical significance, and it is difficult to rank them due to the varying nature of the different outcome variables. However, we considered it more transparent to apply two models. Age, sex, kidney function and diabetes function (and plate number) were thought as variables suitable to adjust for in the first model. In model 2, remaining variables were included, due to their potential influence on the relation between PA and biomarker level.  We have added a short part on model development. | Added sentence on row 282-283 |
|  |  |  |
| Statistical findings are often presented without adequate contextualization. For instance, what are the implications of HDL species being negatively associated with SED? A brief interpretation in the results section could aid clarity. | We agree that there are many relations worth discussing. The implications are many and we have tried to elaborate on this in the discussion. However, the cross-sectional design limits the ability to discuss implications. | Added short sentence on row 453-454 |
|  |  |  |
| The authors mention "robust relations" but do not adequately define how this robustness was assessed beyond statistical significance. | Under “Statistics” we define “robust” relations as those with opposing relations to MVPA vs SED/prolSED and to separate those from biomarkers with relations to only one PA-class. The idea is to separate biomarkers with a more solid (“robust”) and more discrete (“separate”) relation to PA beyond the application of two-sample validation and two different regression models.  We agree that this could be further described, and we have altered the description in the text for clarity. | Added sentence on rows 288-290 |
|  |  |  |
| Table 1: Some demographic variables (e.g., education) are reported without discussing their impact on the study findings. | We thank the reviewer for this comment. The results from the regression analyses including the other independent variables than PA could be presented and discussed. However, to keep the focus on the aim of the paper, we chose to report only the results for the PA classes. |  |
|  |  |  |
| The discussion is overly descriptive and does not sufficiently explore the causal implications of the findings. For instance, are the observed associations indicative of mechanistic pathways or simply correlations? | We agree that the discussion refrains from discussing in causal terms, which is due to the study design. However, the reviewer is correct in that causality could be discussed to a certain degree, based on potential physiological mechanisms. For metabolomic markers, PA is known to increase lipid metabolism and thereby could affect lipid profile via LPL activation or (up-)regulation of LDL receptors. Proteomic markers related to inflammation (such as IL-6) may be affected by PA by alterations to the autonomic system, AMPK activation or changes in for example visceral fat deposits. We have added a few examples, without arguing that we have covered all such mechanisms.  We deemed the number of such mechanisms to be too high to be systematically covered in the discussion. | Added examples of potential physiological pathways on rows 504-510. |
|  |  |  |
| Potential limitations like the cross-sectional nature of the study are mentioned but downplayed. A more critical acknowledgment is warranted, particularly around confounding and selection biases. | We agree with the reviewer that the cross-sectional design prevents any causal inferences, and we have mentioned this throughout the manuscript. We have added a section on selection biases and possible residual confounding in the “Strength and limitations”-section. | Added a paragraph on this on rows 546-555 |
|  |  |  |
| No mention is made of generalizability. Since participants are middle-aged Swedes, findings may not apply to diverse populations. | We agree with the reviewer and have added a text on this | Added text on rows 556-558 |
|  |  |  |
| Reviewer #3 |  |  |
| The focus of this study by Ekblom et. al. is to find an association between various intensity PA classes and metabolomic and cardiovascular protein biomarkers in a middle-aged population. The main drawback of this study is that it is not giving any new information except expanding on the previous correlation between these variables. Metabolomic and Proteomic Profiling are done in different conditions and cohort which doesn't give much emphasis to the manuscript. | We agree with the reviewer that previous investigations have described several of these relations. However, we believe that the study including sensor-based PA assessments and cross-validation in a relatively large and heterogenous population has a value in describing the wider patterns of relations. Also, the lack of convincing relationships between prolSED and biomarkers outside of those found for SED is of potential importance for the design of future intervention studies. |  |
|  |  |  |
| Reviewer #4 |  |  |
| I am grateful for the opportunity to review this manuscript, which examined the associations between accelerometer-assessed physical activity behaviours and CVD-related metabolomic and proteomic biomarkers using two cross-sectional data from the main SCAPIS cohort (discovery data) and SCAPIS pilot cohort (validation data). Though the manuscript is well-written and contains some interesting findings, a few issues may have impacted the scientific quality of the findings. Below are some suggestions/comments/questions the authors may consider if they find them intuitive to enhance the quality of the paper. | We thank the reviewer for critically assessing our manuscript and the possibility to revise in accordance with the points and issues raised. |  |
|  |  |  |
| The authors could briefly describe the demographic characteristics of the studied participants, both the discovery and validated samples. | We agree with the reviewer and have added brief characteristics. | Added data on sex and age in abstract on rows 82-84. |
|  |  |  |
| The number of the main SCAPIS cohort (discovery = 5557) does not match the description in the main text – discovery = 4647) | We thank the reviewer for detecting this, a typo. | Adjusted n in that cohort to n=4647. |
|  |  |  |
| The authors can consider mentioning the analytical method used to examine the associations in the abstract. | We agree that this can increase clarity. | Added information on the statistical method used on row 90. |
|  |  |  |
| The description of the observed associations is unclear, and the authors can help the readers by clarifying the metabolic and proteomic biomarkers and the direction of their associations with the physical activity behaviour classes, especially for the proteomic markers (lines 90 – 92). | We agree that a more detailed description on direction of relations should have been added for proteomic markers as well in addition to those given for the metabolic markers. We also write the full names of the biomarkers, to improve readability. | Altered description in the end of the results section on the manuscript on rows 97-102. |
|  |  |  |
| The authors made several factual statements in the first four opening sentences (page 5, lines 100 – 104) and it would be more appropriate for them to provide some supporting citations (references). | We agree with the reviewer that references are lacking. | Added references and slightly modified sentence on rows 106-107. |
|  |  |  |
| Similarly, the sentences on page 5 lines 110 – 114 may need some supporting references. | We agree that this could be better referenced. Thanks to the comment, we also noted that the section could be more clearly written and have accordingly changed the wording. | Added references and altered text in the introduction. |
|  |  |  |
| Though the introduction looks well-written, it lacks some relevant and specific details that justify the authors’ decision to examine the relationships of physical activity behaviour classes with metabolic and proteomic biomarkers. There is extensive evidence of the associations between the physical activity behaviour spectrum and biomarkers. A brief contextual review of the evidence on moderate-to-vigorous intensity physical activity (MVPA), light-intensity physical activity (LIPA) and sedentary behaviour (SB) (including SB bouts), and the interdependence of these behaviours, would have been appropriate to build their study rationale. | We agree with the reviewer and have added a paragraph on this. The area is large and growing, and we make no attempt to present a systematic overview when presenting data on the associations between PA classes and metabolomic and proteomic biomarkers. | Added references and text on rows 138-151. |
|  |  |  |
| The description on page 9, lines 181 – 184 is a little confusing. The authors may consider clarifying the total number of discovery samples from the regions/sites (5075) and the initial number of included subjects (30154) and after excluding those with missing data the total number of remaining subjects (4887). | We apologize for this inconsistency; it was a typo. The included number was 4647. To further clarify, selection was performed by random numbers | We corrected this in the manuscript. |
|  |  |  |
| It is not clear what “FA variables” means. It may presumably be a typo (PA – physical activity); if it isn’t, it would be appropriate to define abbreviations when used for the first time in the document. | Unfortunately, another typo, should be PA. | Altered in the manuscript. |
|  |  |  |
| It would be appropriate to define the abbreviation “E-GFR”. | The reviewer is correct in that we have not properly defined E-GFR as an estimated glomerular filtration rate. This has now been added. | Added definition on rows 238-240. |
|  |  |  |
| Some readers may also be interested in how the biomarkers used in the Cockcroft-Gault Equation to measure the estimated glomerular filtration rate (eGFR) were assessed | The Cockcroft-Gault formula is based on creatinine. | Added to text on rows 238-240. |
|  |  |  |
| The analysis and results are complex, and the descriptions are comprehensive. There are a few instances, though, where the authors would have been more specific about the direction of the associations (beneficial ‘+’ or detrimental ‘-’) of the physical activity behaviours with the biomarkers. For instance, the description of the associations on page 27, line 298. | We agree that the direction of relationship should have been explained here, and we have added this. We have also scanned the results section for similar mistakes without finding obvious instances for adjustments. | Added direction of relationships in the indicated rows. |
|  |  |  |
| The sentence “Opposite relations were found for ITGB2 and LPL” on page 27, line 316 needs clarity. | We agree that this was not stated clearly and have amended the sentence to be easier to understand | Changed sentence on rows 363-364. |
|  |  |  |
| It is impressive that the authors also explored the potential non-linear associations; however, describing the non-linear analytic approach utilised in the statistical analysis section would be more appropriate. | We agree with the reviewer that this was not described well We have added a description under statistics in the methods section. | “Potential non-linearity for the association between significant biomarkers and PA classes MVPA and SED was explored using a second-degree term to model 2” added on rows 294-295. |
|  |  |  |
| Did the authors also examine the non-linear associations with the metabolic/proteomic biomarkers of LIPA and prolonged sedentary behaviour? | No. As no meaningful non-linear relations were found for SED or MVPA, we did not further progress with these analyses. |  |
|  |  |  |
| There seem to be gender differences in the associations, what is not clear, though, is the direction of the associations. The authors may consider describing the direction of the physical activity behaviours and sex interaction associations with the biomarkers. | We agree with the reviewer that these results are not clearly reported. While all data on these separate relations between biomarkers and PA classes per sex are given in supplementary table 1 and we have added a brief description of these sex differences for clarity. | Added description on direction relations for men and women on rows 381-384. |
|  |  |  |
| Similarly, the authors could describe the direction of the physical activity behaviours and waist circumference interaction associations with the biomarkers. | Again, we agree with the reviewer and have made amendments accordingly. | Added description on direction relations for High and Low WC on rows 389-390 and 396 -397. |
|  |  |  |
| . Did the authors also examine the interaction of LIPA with sex or waist circumference? | No, as the decision to leave LIPA out was made, we did not pursue any such analyses |  |
|  |  |  |
| . Association tables: the authors may have a specific reason to only report coefficients. However, reporting coefficients with confidence intervals or standard errors would be more informative in understanding the statistical significance of the findings. | We agree that adding CIs or measures of variance would give more information. However, the tables are rather large as is and contain only the significant relations. Confidence intervals would all exclude “zero”. The magnitude of for example standard deviations would be small and difficult to read. Therefore, we chose not to include these data. |  |
|  |  |  |
| The findings described in the results mainly focus on the directions rather than the magnitudes/strengths of the associations, however, the authors seem to be highlighting the strengths of the associations in their discussion of the findings. | Yes, this is true. While all reported values are significant, we briefly mentioned the strongest relation in the results section. Further, we made an attempt to stress or put light on the ones with the highest nominal strength in the discussion. We hope that readers will find both informative. |  |
|  |  |  |
| . The authors did not adequately justify why LIPA was excluded from the diagram. As the authors noted in the results section LIPA and sedentary behaviour have a strong negative interrelation, some readers may find it informative if all the physical activity behaviours, including LIPA, are shown in the vain diagram with the intersections. | As stated above, the strong negative relationship (rho=-0.94) between SED and LIPA led us to decide to focus on the three other PA classes. Relations to biomarkers were very similar, but inversed and thus did not add much information. However, we have added a Venn-diagram in the supplementary material, as supplementary figures 3 and 4. The description of the number of unique relations between biomarkers and LIPA has been altered as no biomarker was uniquely related to LIPA (previously stated as one). | Added two Venn-diagrams as supplementary data (Supplementary figures 3 and 4) and altered text in results on rows 396-397. |
|  |  |  |
| The authors could consider a brief description of the vain diagram, highlighting the intersections in the legend. | Figure legends are located in the manuscript and briefly describe the Venn-diagrams. We have added a shorter addition to these legends. | Added text to figure legends. |
