# Supplementary material for: Associations between physical activity and CVD-related metabolomic and proteomic biomarkers
Source: PLoS One. 2025 Jun 11;20(6):e0325720. doi: 10.1371/journal.pone.0325720 (PMC12157240; doi:10.1371/journal.pone.0325720)
Supplement: Attachment — Submitted filename: Response to reviewer250430.docx [file pone.0325720.s013.docx]

Reviewer #4:

1. The authors did a great job satisfactorily addressing most of the concerns; however, their response to one of the comments raises a major concern about the credibility of the reported findings. The reported effect sizes look rather very small. The tables do not look that huge, and the authors may consider restructuring the tables. For them to only report the significant findings without confidence intervals or standard errors may indicate that the authors were selective in their reporting and compromised the validity of the findings.
2. The question, though, is why the authors did not report non-significant results.
3. In some points, the authors also highlight the strength of the associations in their discussion, which is not obvious in the reported coefficients without confidence intervals. The comment with the response is below:

[Citing earlier conversations]

*Comment: Association tables: the authors may have a specific reason to only report coefficients. However, reporting coefficients with confidence intervals or standard errors would be more informative in understanding the statistical significance of the findings.
Response: We agree that adding CIs or measures of variance would give more information. However, the tables are rather large as is and contain only the significant relations. Confidence intervals would all exclude “zero”. The magnitude of for example standard deviations would be small and difficult to read. Therefore, we chose not to include these data.*

Author response:

1. We agree with the reviewer that the coefficients are nominally low, which was mentioned in the manuscript. Based on the suggestion from the reviewer, we have added SE to tables 2, 3, and 4, which may help in interpreting the values.
2. We agree with the reviewer that it is essential to report null findings in scientific reports and are therefore listing biomarkers with non-significant relations to the different PA classes in supplementary table 5.
3. The addition of SEs to the tables may help interpretation in this instance. However, we regarded all significant relations as essential and chose to mention the significant biomarkers with the nominally largest coefficient instead of, for example, those with the lowest p-value. Thus note, all markers in tables 2-4 are significant.
